# The ‘PRICE’ of Physical Activity Referral Schemes (PARS): Stakeholders’ Recommendations for Delivering Quality Care to Patients

**DOI:** 10.3390/ijerph18168627

**Published:** 2021-08-15

**Authors:** Francis A. Albert, Aduli E. O. Malau-Aduli, Melissa J. Crowe, Bunmi S. Malau-Aduli

**Affiliations:** 1 College of Medicine and Dentistry, James Cook University, Townsville, QLD 4811, Australia; bunmi.malauaduli@jcu.edu.au; 2 College of Public Health, Medical and Veterinary Sciences, James Cook University, Townsville, QLD 4811, Australia; aduli.malauaduli@jcu.edu.au; 3 Division of Tropical Health and Medicine, James Cook University, Townsville, QLD 4811, Australia; melissa.crowe@jcu.edu.au

**Keywords:** physical activity, referral schemes, qualitative method, quality of care, healthcare professionals, patients, quality of care model

## Abstract

Evidence-based strategies are needed to curb the growing cases of physical inactivity related morbidities. Delivering holistic care through collaborative shared decision making could boost the effectiveness of physical activity referral schemes (PARS) and foster the quality of care for patients with multimorbidity. A qualitative study involving semi-structured telephone interviews was utilised to gain insights from Australian PARS stakeholders (general practitioners, exercise physiologists, and patients). A pluralistic evaluation approach was employed to explore and integrate participants’ opinions and experiences of PARS and their recommendations were used to develop a model for quality care delivery in PARS initiatives. Five overarching themes: promote, relate, incentivise, communicate, and educate were identified as the ‘PRICE’ for developing effective and functional PARS programmes that foster quality patient care. It was evident that PARS programmes or policies aimed at optimising publicity, encouraging incentives, improving interdisciplinary information sharing and professional relationships between patients and healthcare professionals can transform healthcare delivery and provide top quality PARS care services to patients. Therefore, governments, healthcare systems, and PARS administrators can translate and leverage the insights from this study to optimise the delivery of high quality care to PARS patients.

## 1. Introduction

Healthcare delivery models and policies need to be updated to meet the growing morbidity rate [1] and trends in healthcare systems [2,3,4]. Numerous studies have been conducted to assess the quality of care delivered by healthcare organisations [4,5,6,7,8,9,10]. Abundant evidence supports the exploration of physical activity (PA) as a therapeutic strategy for the prevention, treatment, and management of morbidities (including some cancers) and mortalities in various settings [11,12,13]. Morbidities and mortalities could be reduced by promoting PA interventions, such as brief advice, counselling, and collaborative care through onward exercise referral (patient care transition from frontline primary care professionals, such as general practitioners (GPs) to PA specialists, e.g., exercise physiologists (EPs) [14,15]. 

Collaboration via GP to EP referrals would be invaluable in developed countries, such as Australia, where nine out of ten patients see a GP at least once a year [16,17]. This highlights the enormous potential of leveraging the access of frontline healthcare professionals (HCPs), such as GPs, as gatekeepers and vanguards of PA promotion to the population [18]. The efficiency and long-term sustainability of these primary care interventions are, however, fraught with doubts due to obstacles, such as the lack of time, adequate skill, and knowledge to promote PA by frontline HCPs [19,20], and low patient referrals from healthcare gatekeepers, such as GPs to PA specialists (e.g., EPs) [21]. Given that patients with multimorbidity require long-term quality care from different HCPs [22], the current healthcare service delivery structure might struggle to provide optimum and quality healthcare services to these patients [23]. This necessitates a paradigm shift in healthcare systems towards delivering sustainable and efficient chronic disease management interventions [24]. 

Collaborative shared decision making (a team care approach where the care provided to a patient by a group of HCPs reflects the values and choice of the patient) [25] could foster the delivery of quality care to patients and enhance their health outcomes [26]. Delivering high quality care to patients could improve wellbeing and quality of life, optimise the quality of healthcare service delivery, and reduce hospital admissions [27]. The quality of healthcare initiatives are constantly evolving, and the evidence in support of current strategies are inconclusive [28,29,30]. For example, previous studies examining the quality of care have primarily focused on patient satisfaction and are now shifting towards patient experiences [30,31]. Furthermore, current studies advocate for evidence-based, meaningful, and consistent interactions between healthcare professionals and patients [24,32].

However, research into the factors that foster the promotion of quality healthcare to patients is scarce, particularly regarding PARS interventions. Thus, employing a pluralistic approach to explore the views of key PARS stakeholders (GPs, EPs, and patients) would help inform the development of policies that could foster quality care delivery and boost the effectiveness of the PARS programme [33]. Recommendations of key PARS stakeholders, such as GPs, EPs, and patients on how to promote the PARS programme in an Australian context within one study has not been previously explored. Thus, this qualitative study aimed to fill this research gap by empirically exploring the views of GPs, EPs, and patients on the quality of care in PARS referrals. It also aimed to substantiate the evidence base and inform a quality of care model that could optimise healthcare delivery to patients for improved health outcomes and PARS effectiveness.

This study was guided by the research question: What are participants’ (GPs, EPs, and patients) views on how to optimise the quality of care in PARS referrals to enhance PA and patient health outcomes? It is hypothesised that insights gained from the views of stakeholders will assist to inform policies for an effective PARS programme and healthcare delivery.

## 2. Methods

### 2.1. Study Design

A qualitative study design guided by the tenets of the consolidated criteria for reporting qualitative studies (COREQ) guidelines [34] (Appendix A) and pluralistic evaluation [33] approach was employed to explore the opinions and experiences of the PARS stakeholder groups (GPs, EPs, and patients). The pluralistic approach involved synthesising PARS stakeholders’ views to reach a consensus on the best approach to promoting quality care in PARS referrals [35]. 

### 2.2. Participants

Participants included Australian HCPs (registered GPs and EPs) and patients who have used PARS services. Respondents were 18 years and above and based in Australia at the time of this study. A purposive sampling strategy (non-random identification and selection of suitable study participants) was used to recruit the participants for this study. This technique included: (1) identification of participants who were a representative sample of the population via a pre-interview survey [36,37]; (2) purposively selecting and contacting respondents who could provide valuable information and represent heterogeneity in the population; and (3) acknowledgment of consent and commitment by participants to take part in the interview. 

### 2.3. Data Collection

To understand HCPs’ and patients’ views and experiences of quality care in PARS initiatives, semi-structured individual telephone interviews of approximately 40 minutes duration with GPs, EPs, and patients were conducted and audio taped. A semi-structured interview approach was used to allow the interviewer prepare questions beforehand to help guide the conversation and allow for more in-depth focused discussion on the topic [38]. The telephone was used because of its flexibility and access to respondents across the country [39]. Interview questions (10 semi-structured questions) were developed based on findings from previous PARS studies [36,37] and pilot tested on eight participants (two GPs and three each of EPs and patients) by the primary researcher (FAA) and reviewed by BSMA to test the usability and credibility of the interview questions. In addition, the findings from the pilot interviews were used to refine the final interview questions.

The interviews were conducted between August and December 2020, and each interview began with an acknowledgement of consent and concluded with a summary of interview accounts with respondents to facilitate transparency and shared understanding. Major areas of exploration in relation to this study were participants’ experiences of PARS and their recommendations to foster an improvement of the programme. Follow-up probes and prompts were used to encourage further insights into respondents’ views and experiences. Interviews were stopped when data reached saturation (when no new information enhanced the researchers’ understanding of quality care in PARS referrals) [40].

### 2.4. Data Analysis

Audio-recorded interviews were transcribed verbatim by F.A.A. identifying information removed and pseudo-names assigned to quotes. Pseudo-names beginning with Dr were given to GPs, ending with EP to EPs and none for patients. Transcribed interview data were imported into NVivo software version 12 (QSR International Pty Ltd. Victoria, Australia: 2018) for data storage, management, and analysis [41]. Attride-Stirling’s [42] inductive thematic analysis principles were used to analyse the interview data. This process included (1) familiarisation with the interview transcripts to identify codes; (2) grouping of codes into themes based on their commonalities; (3) grouping of themes into thematic networks based on their conceptual content; (4) further exploration of thematic networks for cause and effect relationships; (5) development of a model linking the conceptual findings in the thematic network to the research question.

Data transcription, coding, and theme generation were independently conducted and confirmed by F.A.A. and B.S.M.-A. Discrepancies were resolved in a consensus meeting.

### 2.5. Ethical Consideration

This study was approved by James Cook University’s (JCU) Human Research Ethics Committee (HREC) (reference number: H7661). Prior to participating in the study, participants were furnished with detailed information about the study, and they were required to provide consent.

## 3. Results

Forty (40) respondents, including GPs (*n* = 8; 0% female), patients (*n* = 15; 80% female), and EPs (*n* = 17; 65% female) took part in this study. Participants’ average ages were 44 years for GPs, 31 years for EPs, and 61 years for patients. All GPs indicated they worked in private practice with an average work experience of 13 years. EPs had an average work experience of 7 years, and all except three of the EPs worked in private practice. Two out of these three EPs noted their practice as a teaching setting (university), while the remaining EP worked with a non-governmental organisation (NGO). The main reasons patients gave for their referral to PARS included diabetes, stroke, chronic back pain, and overweight/obesity. 

**Figure 1:**
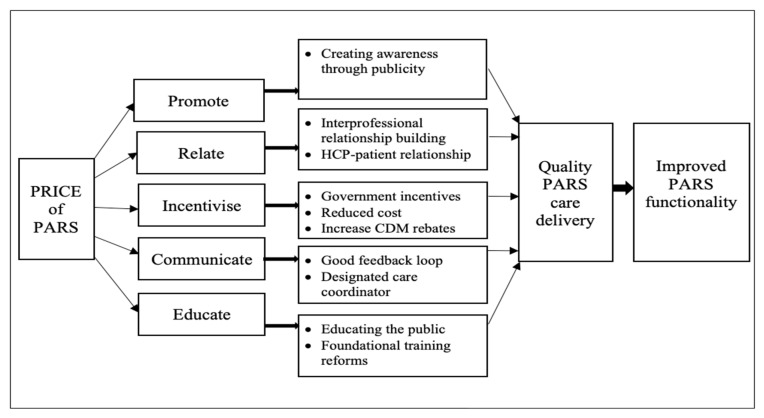
A model for promoting quality care in PARS referrals.

Five overarching themes and 10 sub-themes emerged from this study. They include promote (sub-theme: creating awareness through publicity), relate (sub-themes: interprofessional relationship building and HCP–patients relationship), incentivise (sub-themes: government incentives, reduced cost, and increase chronic disease management (CDM) rebates), communicate (sub-themes: good feedback loop and designated care coordinator), and educate (sub-themes: educating the public and foundational training reforms. Based on the study findings, a model is presented for fostering effective PARS referrals and promoting quality care for PARS patients, see Figure 1. 

### 3.1. Promote

Participants perceived that the direct promotion of PA and PARS information by HCPs would foster the functionality of the PARS process and enhance the delivery of quality care to patients. Respondents’ perceptions regarding how to promote PA and the PARS programme were categorised into the sub-theme: creating awareness through publicity.

*Creating awareness through publicity:* all participants identified the promotion of PA and PARS as an important initial step in improving quality of care for patients. They recommended the use of avenues, such as information sessions, campaigns, and media to promote the programme.

GPs urged EPs to use forums, such as information sessions to inform the public about distinctions between their roles and other allied health professionals. Furthermore, GPs suggested the need to improve the media promotion of EP services through multiple channels.

*“The exercise physiologist has to do a lot of campaigns to convince people how their service is probably different from that of a chiropractor or a physiotherapist. Media coverage is also important. I do see that some of the exercise physiologists in this town place some adverts on the television. They should also do broadcasts through the radio stations to enlighten the community more about what they stand to gain from such exercise referrals. I think enlightenment is very important” Dr KC 42.*

EPs corroborated the views of the GPs by saying that the dissemination of PARS information could help enlighten the public on the benefits of taking up PA interventional programmes, improve awareness about the roles and services EPs provide and help patients seek referrals themselves.

*“An awareness campaign to the general public could be quite beneficial, because if you are getting more people aware of the system, they’re going to come in and ask the doctor about it without their doctor having to bring it up” JT 26 (EP).*

In addition, EPs indicated that GPs’ awareness of the roles and services they [EPs] render is critical to the programme’s success.

*“When the GPs have more of an understanding about what we do and how to talk to patients about it, we get better success rates and people taking up that kind of programme” LR 28 (EP).*

Some patients supported this notion by suggesting that information about the services of EPs be made readily available in the community, particularly in key healthcare centres (e.g., hospitals).

*“I think really [it is about] information, even if as I was discharged, there was a brochure for an exercise physiologist, … as you know these are the things that you might want to follow up” NB 41.*

Other patients further added that making promotional materials, such as pamphlets, available to GPs could help the doctors promote the programme better.

*“What I will say is leaflets, like good quality advertising pamphlets sent to GPs that they could put in their waiting rooms. I think it’s that kind of stuff—Look, I do want to give patients something, what can I give them; Oh, hang on there is a pamphlet here” SM 63.*

### 3.2. Relate

Participants regarded the building of successful interprofessional relationships among HCPs as well as patient-HCP relationship as key determinants of quality healthcare delivery to patients. This would in turn enhance the functionality of the PARS programme. Respondents’ comments on how to relate were categorised into the subthemes of interprofessional relationship building and HCP–patient relationships.

#### 3.2.1. Interprofessional Relationship Building

Participants perceived that developing respectful and efficient interprofessional relationship among HCPs could foster information sharing and improve the quality of care for patients.

GPs voiced that a consistent engagement between them and EPs could advance insights into available EP services.

*“If we see them, if we talk to them. Usually, why do you think the drug reps come to see us almost every week. The closer they are to us, the more they remind us of what they sell. If the exercise physiologists come to see us, even if it is once a month, one way or the other, they will answer questions, they will provide solutions and some advice on what they could offer and what is available” Dr CL 44.*

EPs echoed the views of the GPs by saying that engaging with GPs would make it easier for the doctors to refer patients for PARS interventions and facilitate the exchange of supporting materials that could ease the referral process.

*“If you build a relationship with the GP, that GP is probably going to refer to you because it’s easy to do so. So, we need to make it easy for GPs to refer in the first place.” LB 34 (EP).*

Patients endorsed the views of the HCPs by advocating for stronger ties between GPs and EPs.

*“I think you have to address that issue, which is my personal experience. There is a break down [in communication] between the GP and the EPs” LD 68.*

#### 3.2.2. HCP–Patient Relationships

Rapport building between HCPs and patients was viewed by participants as pivotal to improving the functionality of PARS. GPs felt that spending more time with patients could help them better promote PA to the patient.

*“If one could have an opportunity to have more time with patients. I think it would go a long way in improving the delivery of PA information to the patient” Dr CF 43.*

EPs substantiated the views of the GPs by noting that collaboration between a GP and an EP who share a common goal would enhance quality PA and PARS care delivery to patients.

*“The biggest thing that I’ve learnt in practice is finding that key GP. Someone who is as motivated as you are, who is as passionate as you are and is really willing to take time out of their day. And you’re willing to take time out of your day for the patient care” ER 26 (EP).*

Patients emphasised the importance of rapport building between HCPs and patients as this is essential for patient uptake and adherence to recommended PA and PARS.

*“Well, it depends, we can be referred to these things, we can talk to the referred person right, but if there’s no connection between that person and you yet again, you won’t do anything. If there was a connection then, that becomes a different thing…there’s got to be something there to make you want to do it” BR 65.*

### 3.3. Incentivise

There was consensus among respondents on the need to use incentives as a strategy to facilitate HCPs’ provision of quality care in PA and PARS to enhance uptake and adherence to intervention goals by patients. Participants’ recommendations on incentives were categorised into three sub-themes including government incentives, reduced cost and increase CDM rebates.

#### 3.3.1. Government Incentives

Respondents urged the government to review currently available incentives to intensify efficient delivery of PARS.

GPs argued for increases in payment as an incentive for coordinating PARS.

*“Government can increase the payments to the GPs as incentive to coordinate patients’ care plan and team care arrangements and the referrals” Dr GE 44.*

EPs and patients supported this notion and emphasised the importance of holistic approach to healthcare delivery.

*“There should be more emphasis on GPs. We should probably actually think about prevention and actually incentivising GPs to make these kinds of referrals” LB 34 (EP).*

*“The government should make it financially worthwhile for GPs to actually do what most of them want to do and that is manage all of this care and to coordinate it all and to look at a person’s overall health file rather than just the acute things that they come in with” DM 70.*

#### 3.3.2. Reduced Cost

Participants reported cost as a barrier to HCPs coordinating PARS care for clients and patients’ uptake of PA and PARS initiatives. 

GPs urged the government to subsidise PA and PARS intervention cost for patients, particularly the elderly, because of the positive effects of the interventions on their wellbeing.

*“The government should also throw more weight in terms of subsidising the costs of people gaining access especially for the elderly. I find them to benefit more because they have to do some balance and stability training” Dr KC 42.*

EPs and patients reiterated the burden of cost challenges expressed by GPs. EPs suggested the delivery of affordable care by specialists.

*“Cost is quite something you know, it’s one prohibitor of people attending services. So, you know, referral schemes are really helpful in how you provide your service to minimise, to reduce the cost to the client is important” LS 35 (EP).*

Patients advocated for cost subsidies to help patients afford the preventative benefits of the programme, rather than paying a huge cost to seek an overdue solution.

*“Another big piece of this problem is the economic issue. Lots of people are unable to afford that. So, for that, government should really do a bit more for they call it preventive methods, because they spend so much money on the medical side, but that’s too late when they are sent to the hospital, it’s too late. So, it’s a big gap” LD 68.*

#### 3.3.3. Increase CDM Rebates

Participants argued that the current five CDM rebate-able sessions provided by Medicare are inadequate. 

GPs proposed a refinement of the number of free CDM sessions allocated to patients per year. They suggested an increase from five to 10 sessions per year.

*“Medicare reviewing the enhanced primary care [EPC] pathway and see if it’s possible to increase the number of referrals yearly, may be from five to maybe about 10. That will be one way that it could be improved” Dr ON 52.*

EPs substantiated the perspectives of the GPs and argued against setting a limit for the number of free sessions at the beginning of PA and PARS interventions to allow specialist enough sessions for behavioural change.

*“I think it’s important that we always focus on getting someone independent and I think that’s the idea as five sessions only is to stop seeing people when they probably don’t need it. So there needs to be things in place to stop people doing that, but I feel that in the first, two, three months, that’s really critical for behaviour change. And if we can just get more sessions in that time and then get less for the rest of the year just to monitor them and make sure they’re keeping on top of everything. I think that will be a better approach and they’ll be more successful” LS 35 (EP).*

Patients supported the views of the HCPs and called for extra free sessions to help maximise the gains of PA and PARS interventions.

*“We need more of that and that was the whole idea of doing this interview with you, there needs to be more. If people want to fight the obesity, if they want to fight the diabetes that goes along with that, then people who need it, should get to it without a great expense” DM 70.*

### 3.4. Communicate

Clear and effective communication among HCPs and between HCPs and patients were viewed as vital to achieving success in delivering quality care in PA and PARS services. Respondents’ suggestions on ways to communicate were summarised into two sub themes including good feedback loop and designated care coordinator.

#### 3.4.1. Good Feedback Loop

Participants’ views regarding efficient two-way communication show the importance of maintaining an efficient feedback loop among HCPs.

GPs emphasised the need to maintain a good information exchange channel to help them keep up to date with the care of the patients they referred into PA and PARS programmes.

*“A lot of times you don’t hear anything from the EP so you are kind of in the dark in terms of what is happening and because you see many patients you might not even keep track of the patient you refer to the EP if you don’t hear from them” Dr BO 40.*

EPs substantiated the views of the GPs and suggested an overview of current communication pathways to include useful tools, such as templates to help guide the information exchange between them and frontline HCPs (e.g., GPs).

*“Communication channels need to be refined between the two. So there’s specific templates that go back and forth that are more detailed in nature. So there’s an expectation from the GPs that the goals are more specific [and reported] in a measured, smarter way essentially, so the practitioner knows what they’re going to be dealing with. The GPs should expect a more detailed report that actually stipulates what assessments they did and what they found from those assessments and potentially the plan moving forward” LB 34 (EP).*

Patients recommended that frontline HCPs, such as GPs, should be constantly reminded of available EP services and provided with printed information to be disseminated to their patients.

*“They need to be reminded constantly and given something like hardcopy [information] for their patients, not just on an e-mail or something, because they’ll forget about it” SM 63.*

#### 3.4.2. Designated Care Coordinator

Participants reported similar views regarding the nomination of a specialist HCP whose primary duty will be to coordinate PARS for patients. All respondents nominated a nurse as the best suited HCP for that role.

GPs supported their choice of a nurse with a view that nurses can make out time for providing quality care for patients involved in PARS initiatives.

*“If the patient liaises with the practice nurse in the preparation of the chronic disease plan, the patient can be educated more. The nurse has more time to discuss further with the patients and answer all the questions thereby increasing compliance on the side of the patient” Dr GE 44.*

EPs echoed the thoughts of the GPs and felt that nurses are the largest homogenous group of HCPs in the hospital and they are vital for improved functionality of PARS.

*“Nursing staff mainly because of the fact that they are the biggest proportion in the hospitals because nurses are also a key to initiate referrals” LS 35 (EP).*

Patients endorsed the views of the HCPs and argued that nominating a particular HCP, such as a nurse as a PA and PARS expert, would help them coordinate effective and quality care during PA referrals. They also perceived that it would relieve them [patients] of the burden of coordinating their own care.

*“So, it would have been good to have someone sort of coordinating all this, even a nurse or an allied health professional or someone that was like a coordinator rather than leaving the burden with me to sort of keep on top of it. Because I’ve got all these conditions and it’s hard to keep track of them all, even though I know what I’m doing and that caused me more stress” RS 65.*

### 3.5. Educate

Participants perceived education as a vital tool for informing quality care delivery and suggested ways to go about it. Respondents’ perspectives on how to educate the population about PARS were grouped into two sub-themes (educating the public and foundational training reforms).

#### 3.5.1. Educating the Public

There was consonance between respondents’ views regarding the need to enlighten the public particularly, frontline HCPs, such as GPs, on the value, role, and availability of PA and PARS services, and how to deliver quality care for patients. 

GPs proposed a general orientation on the services provided by EPs. They urged that relevant stakeholders’ knowledge about the roles of EPs could be enhanced, particularly through media channels, such as television and the internet.

*“I recommend better education, on the side of the GPs about exercise physiologists. Again, education or mass orientation. The department of health could do a good job by letting the people know out there, that supervised exercise regimen is necessary for the treatment of many chronic disease conditions, in fact in the form of social mobilisation, online, TV and the rest of them” Dr GE 44.*

EPs substantiated the views of the GPs by suggesting that PA and PARS education for frontline HCPs, such as GPs, be incentivised to make it worthwhile for the gatekeepers.

*“GP education is a big one, but you’ve got to make it so they are actually getting something out of it. So rather than it being just like disbursed information and then it’s up to them to follow up on it; GPs should be allowed to use that as a continuing professional development point, so that they’re having incentive to do it. They’re so busy all of the time, you can give them extra work that they’re not being paid to do, if they are not getting anything out of it, they are just not going to do it” AN 31 (EP).*

Patients suggested that GPs may not be fully aware of the promotional incentives provided by the government. They proposed more educational/awareness programmes to help GPs promote the initiative effectively.

*“I’ve only just found out that the government is subsidising some of these things, but I don’t know whether that’s new or whether that has been around. From my point of view, it would be really worthwhile for people like myself to know that is available, particularly for pensioners or people with lower income to be able to access these things. So, if the GPs were more aware of that, too, they might even recommend it” LR 61.*

#### 3.5.2. Foundational Training Reforms

Participants proposed the inclusion of PA and PARS training in the curriculum of prospective medical graduates.

GPs felt that being knowledgeable about interventions that could be useful to their patients and implementing them would be invaluable to their practice.

*“It is about the GPs being knowledgeable in what will help their patient in certain conditions and be able to implement that” Dr CL 44.*

EPs argued that including PA and PARS information into the medical curriculum would help GPs to effectively deliver quality PA and PARS care to their patients.

*“My idea will be to educate the next generation of GPs coming through, so they are in university, explaining what our services are and how it can help their clients” SM 22 (EP).*

Patients corroborated these views.

*“It needs to start within the university medicine programmes around the country. It almost looks like we just have to wait it out and as more graduates come through and get into practice, then things will start to change” NB 41.*

## 4. Discussion

This qualitative study explored the recommendations of key PARS stakeholders (GPs, EPs, and patients) on PA and PARS and developed a model for improving the functionality of PARS to ensure delivery of quality care to PARS patients. The findings revealed that education about and promotion of PARS services, ongoing interprofessional collaboration, HCP–patient relationship building, and proper incentivising are critical to delivering quality care through PARS. These participants’ recommendations reinforce the need for reforms in healthcare delivery policies that foster financial support from government, innovative patient engagement and HCP interprofessional collaborative care [43,44,45]. 

Ongoing interactions, exchange, and promotion of useful information about PARS among HCPs were perceived as crucial for improved PARS functionality and a conduit for delivering quality care to users. Sustained information sharing culture among HCPs could help frontline HCPs such as GPs, to be up to date with PA and PARS information and provide motivation to recommend it to their clients when needed. Therefore, mass promotion of PARS initiatives via primary healthcare interventions supported with printed materials such as pamphlets and diverse media publicity platforms, could enhance the effectiveness of the PARS programme and provide further insights into the roles, benefits, and availability of EP services [37]. Participants also proposed nominating designated PA and PARS specialist in healthcare centres to support GPs, in promoting and coordinating quality care for PARS users. In light of this, nominating other HCPs such as nurses, to coordinate quality care for PARS participants could foster the programme’s uptake and ease the extra burden on GPs [46,47]. 

Respondents perceived that the development and nurturing of interprofessional and HCP–patient relationships could boost the gains made from the PARS initiative and improve quality care delivery for the programme’s users. Strong interprofessional collaborations and HCP–patient interactions through shared decision making could promote trust, confidence in the use of EP services and strengthen patients’ perception of quality care [26]. For example, a six-month intervention that included education workshops to increase teamwork among HCPs in 26 general practices enhanced professional collaboration among HCPs and improved patients‘ involvement and empowerment in the care process [48]. Furthermore, enabling a multidisciplinary care approach among frontline HCPs such as GPs and allied health professionals, particularly EPs, could enhance quality of care delivery to patients and increase positive behavioural change towards PA and PARS interventions [49,50]. Respondents believed that incentives from the government could enhance patient access and affordability of PARS initiatives and boost the delivery of quality care for the programme’s users. Therefore, an efficient use of incentives to promote PA and PARS initiatives could enhance the delivery of quality care in PARS, increase the programme’s usage and potentially enhance patient health outcomes [51,52].

Recommendations by participants to educate the general population on PARS initiatives, implied that they perceived education as the bedrock for building a solid foundation for quality care delivery in PARS. It also suggests the lack of general understanding of EPs’ roles in the Australian healthcare system, both by other HCPs and the public. Participants proposed a continuing professional development reward system for GPs to help them see the value of engaging with new knowledge about PA. In addition, they perceived the enlightenment of community members to be critical to the uptake and functionality of the PARS programme. Some participants suggested the inclusion of PA and PARS training programmes as components of the medical education curriculum to help doctors gain insights into various intervention strategies including those of PA that could assist them to provide optimal care to patients. Reforms or policies that encourage frontline HCPs such as GPs, to seek PA and PARS knowledge could be invaluable to delivering quality care to patients and enhance the functionality of the PARS programme [53,54].

### 4.1. Strengths and Limitations

To our knowledge, this is the first qualitative study that explored the voice of key PARS stakeholders to develop a model for the effective use of PARS and the promotion of quality care through the referral pathway. Employing a pluralistic strategy ensured that all participant groups had their views represented in this study. Representing the views of PARS’ main stakeholders further strengthens the evidence in this study. However, considering the perspectives of particular groups of patients and HCPs (GPs and EPs) means that this study did not include other HCPs (such as occupational therapists and physiotherapists) involved in PARS. Additionally, this study’s results should be interpreted with caution because the findings are based on the views of Australian participants, which may not be directly transferable to other settings. Furthermore, participants’ responses, particularly those of HCPs, could have been biased due to their work affiliations and interest in PARS initiatives. 

### 4.2. Implications for Practice and Research

The model developed from this study can be used as a guide for delivering optimum care to patients in PARS interventions. The evidence from this study can be used to support the development of policies and interventions, such as the inclusion of PA promotional information in the curriculum of learners who are training to become doctors. This measure could promote quality PA and PARS care for patients, and ultimately lead to better health outcomes for patients and improve the functionality of the PARS programme. The model could help identify key factors that hamper (e.g., poor feedback) or promote (e.g., incentives or promotions via diverse media outlets and pamphlets) the delivery of effective quality care services in PARS. Furthermore, PARS administrators can leverage participants’ suggestions about better ways to relate (e.g., building rapport), educate (e.g., professional development points) and communicate (such as designating a specialised care coordinator e.g., a nurse) PA and PARS intervention goals to refine or reform programmes that reflect end users’ choices. This will encourage the promotion of quality care and augment the functionality of the PARS programme. Further studies from diverse settings and involving other HCPs on how to effectively promote quality PA and PARS care to patients are needed. This would substantiate the evidence base and provide a clear understanding and consensus on the quality and effectiveness of PA and PARS care delivery across the globe. 

## 5. Conclusions

This study employed a pluralistic approach to explore the views of key PARS stakeholders (GPs, EPs, and patients) to develop a model for promoting quality care in PARS and enhancing the functionality of the referral pathways. Identifying critical quality care constructs is essential to the optimisation of sustainable interventions and programme development. Findings from the study highlighted that, to propagate effectiveness and quality care delivery, PARS administrators need to develop policies that support promotion, communication, and education about PARS services and provide incentives to service providers and users. This approach would promote collaborative care among HCPs, boost the uptake and functionality of the PARS programme and enhance patients’ experiences of quality care and beneficial health outcomes. 

## Data Availability

The dataset supporting the findings in this study are included within the article.

## Abbreviations

CDM	chronic disease management
EPs	exercise physiologists
EPC	enhanced primary care
ESSA	exercise and sports science Australia
GPs	general practitioners
HCPs	healthcare professionals
HDR	higher degree by research
HREC	Human Research Ethics Committee
JCU	James Cook University
PA	physical activity
PARS	physical activity referral scheme

## Appendix A

**Table A1:** Protocol for the above Study Based on the COREQ Checklist. Adapted from: Tong, A.; Sainsbury, P.; Craig, J. Consolidated Criteria for Reporting Qualitative Research (COREQ): A 32-Item Checklist for Interviews and Focus Groups. *International Journal for Quality in Health Care* 2007, *19*, Number 6: pp. 349–357.

No	Item	Description	Reported on Page #
**Domain 1: Research Team and Reflexivity**			
Personal Characteristics			
1.	Interviewer/Facilitator	The primary researcher, F.A.A. conducted the interviews.	5
2.	Credentials	The interviewer (first author—**F.A.A.**) holds a BSc in Human Physiology and an MSc in Exercise and Sports Science; Other Authors: **A.E.O.M.A.**: BSc, MSc, PhD; **M.J.C.**: BSc, GradCert TT, PhD **B.S.M.A.**: BSc, MSc, GradCert ULT, GradCert Mgt, PhD.	Not applicable
3.	Occupation	**F.A.A.** is a PhD higher degree by research student at the College of Medicine and Dentistry, James Cook University Townsville, Queensland, Australia; **A.E.O.M.A.** is an Associate Professor in the College of Public Health, Medical and Veterinary Sciences, James Cook University, Townsville, Queensland, Australia; **M.J.C.** is an Associate Professor at the Division of Tropical Health and Medicine, James Cook University, Townsville, Queensland, Australia; **B.S.M.A.** is an Associate Professor at the College of Medicine and Dentistry, James Cook University, Townsville Queensland, Australia.	21
4.	Gender	By author: F.A.A.: male; M.J.C.: female; A.E.O.M.A.: male; B.S.M.A.: female	Not applicable
5.	Experience and Training	All authors have vast experiences in research and published articles on PARS in peer-reviewed journals.	Not applicable
Relationship with Participants			
6.	Relationship Established	The interviewer had no prior relationship with any of the interviewees.	Not applicable
7.	Participant Knowledge of the Interviewer	Each interview commenced with a verbal acknowledgement of consent. Participants were informed about the objectives of the study during the introductory stages of the interview.	5
8.	Interviewer Characteristics	The interviewer did not report bias of any kind.	Not applicable
**Domain 2: Study Design**			
Theoretical Framework			
9.	Methodological Orientation and Theory	As outlined in the manuscript, inductive thematic analysis was employed in the qualitative phase of the study.	6
10.	Sampling	Participants were purposively selected for the study from a pre-interview survey.	5
11.	Method of Approach	Participants were approached through their email or telephone numbers depending on the contact details they provided.	Not applicable
12.	Sample Size	40 participants (8 GPs, 15 patients, and 17 EPs).	7
13.	Non-Participation	None	
Setting			
14.	Setting of Data Collection	The interviewer was located in a secured office during the interview. Participants acknowledged the comfort of their location before the interview proceeded.	Not applicable
15.	Presence of Non-Participants	No	Not applicable
16.	Description of Sample	Respondents must be 18 years and above and based in Australia at the time of this study and HCPs have to be registered to practice in Australia.	5
Data Collection			
17.	Interview Guide	To understand HCPs’ and patients’ views and experiences of quality care in PARS initiatives, semi-structured individual interviews of approximately 40 minutes duration with GPs, EPs, and patients were conducted and audio taped. A semi-structured interview approach was used to allow participants the liberty to express their views and lived experiences regarding the quality of care in PARS referrals. Data were collected via telephone between August and December 2020. The telephone was used because of its flexibility and access to respondents across the country Interview questions (10 semi-structured questions) were developed based on findings from previous PARS studies and pilot tested on eight participants (two GPs and three each of EPs and patients) by the primary researcher (F.A.A.) and reviewed by B.S.M.A. to test the usability and credibility of the interview questions. Major area of exploration in relation to this study was participants’ experiences of PARS and their recommendations to foster improvement of the programme. F.A.A. conducted interviews, and each began with an acknowledgement of consent and concluded with a summary of interview accounts with respondents to facilitate transparency and shared understanding. Follow-up probes and prompts were used to encourage further insights into respondents’ views and experiences. Interviews were stopped when data reached saturation (when no new information enhanced the researchers understanding of quality care in PARS referrals).	5–6
18.	Repeat Interviews	Repeat interviews were not required.	Not applicable
19.	Audio/Visual Recording	Data were collected using audio recording. Visual recording was not required.	5
20.	Field Notes	None	Not applicable
21.	Duration	Each interview lasted approximately 40 minutes.	5
22.	Data Saturation	Yes—interviews continued until data saturation.	6
23.	Transcripts Returned	No, but the research team checked the interview transcripts for accuracy.	Not applicable
**Domain 3: Analysis and Findings**			
Data Analysis			
24	Number of Data Coders	Two (2), researchers (F.A.A. and B.S.M.A.) independently coded the data and developed and mapped all themes against those of the care coordination model. A series of consensus meeting between FAA and BSMA facilitated the verification of all the codes generated.	6
25	Description of The Coding Tree	Yes, see data analysis.	6
26	Derivation of Themes	All themes were derived inductively from the data.	6
27	Software	QSR international’s NVivo version 12 for Mac was used in the management of the qualitative data.	6
28	Participant Checking	Yes, the interviewer summarised interview accounts with each participant after the interview.	6
Reporting			
29	Quotation Presented	Sample quotes were presented for each of the theme generated.	8–16
30	Data and Findings Consistent	Yes, data and findings were consistent.	Not applicable
31	Clarity of Major Themes	Yes, major themes are clear and presented in the body of the manuscript.	8–16
32	Clarity of Minor Themes	Yes, minor themes are clear and presented in the body of the manuscript.	8–16
